# Mitochondrial DNA: Distribution, Mutations, and Elimination

**DOI:** 10.3390/cells8040379

**Published:** 2019-04-25

**Authors:** Chaojun Yan, Xiaoying Duanmu, Ling Zeng, Bing Liu, Zhiyin Song

**Affiliations:** Hubei Key Laboratory of Cell Homeostasis, College of Life Sciences, Wuhan University, Wuhan 430072, China; ycj114@whu.edu.cn (C.Y.); duanmu@whu.edu.cn (X.D.); zengling368@whu.edu.cn (L.Z.); bingbingliu@whu.edu.cn (B.L.)

**Keywords:** mitochondria, mtDNA, mitophagy, mtDNA distribution, mitochondrial dynamics

## Abstract

Mitochondrion harbors its own DNA (mtDNA), which encodes many critical proteins for the assembly and activity of mitochondrial respiratory complexes. mtDNA is packed by many proteins to form a nucleoid that uniformly distributes within the mitochondrial matrix, which is essential for mitochondrial functions. Defects or mutations of mtDNA result in a range of diseases. Damaged mtDNA could be eliminated by mitophagy, and all paternal mtDNA are degraded by endonuclease G or mitophagy during fertilization. In this review, we describe the role and mechanism of mtDNA distribution and elimination. In particular, we focus on the regulation of paternal mtDNA elimination in the process of fertilization.

## 1. Introduction

Mitochondrion is a double-membrane organelle that generates about 90% of cell energy in the form of adenosine triphosphate (ATP) by the oxidative phosphorylation (OXPHOS) process in mammalian cells. Mitochondria also play an essential role in a series of signal pathways, including tricarboxylic acid cycle (TCA), the β-oxidation of fatty acids, and calcium handling [1], and in regulating intrinsic apoptosis [2,3] and participating in the cell cycle [4,5].

Unlike the other organelles in a mammalian cell, mitochondria have a small amount of their own DNA, which is known as mitochondrial DNA (mtDNA), which encodes a series of crucial proteins for mitochondrial respiration. Each mitochondrion contains one or more copies of mtDNA, which are located in the mitochondrial matrix [6]. Different from nuclear DNA (nDNA), but similar to bacterial chromosome, mtDNA is packaged by a range of proteins including prohibitins, ATPase family AAA domain-containing protein 3 (ATAD3), mitochondrial transcription factor A (TFAM), POLG (DNA polymerase gamma, catalytic subunit), etc., and forms an mtDNA–protein complex, which is called a nucleoid. Among the identified nucleoid proteins, TFAM (mitochondrial transcription factor A) is the main protein of the nucleoid, and acts as a transcription factor of mtDNA in mitochondria and plays an important role in nucleoid distribution and organization [7,8]. mtDNA distributes throughout the mitochondrial network, which is essential for the maintenance of mitochondrial functions. Defects of mtDNA distribution are associated with many human diseases [9,10].

The mtDNA is particularly susceptible to certain stress-induced damages due to a lack of histones in the structure and effective repair mechanisms [11,12]. mtDNA mutation caused by stress-induced damage is highly associated with various human diseases. mtDNA mutation causes damaged and dysfunctional mitochondria, which could be eliminated by mitophagy. The well-known pathway of mitophagy is mediated by the PINK1 (PTEN induced kinase 1)–Parkin pathway [13].

mtDNA is inherited from the maternal line, and paternal mtDNA is degraded during fertilization. How paternal mtDNA is removed in the process of fertilization has always been a critical scientific question. It has been reported that mitophagy and endonuclease G contribute to paternal mtDNA clearance during fertilization [14,15,16,17,18].

Given the role of mtDNA dysfunction in several human diseases, it is important to understand mtDNA distribution and clearance in cells. In this review, we focus on the mechanism of mtDNA distribution, and also discuss the pathways of the paternal mtDNA elimination.

## 2. mtDNA Structure

The structure of mtDNA is significantly different from that of nDNA; however, similar to the bacterial chromosome, mtDNA forms a closed circle doubled-stranded DNA in nearly all metazoa [19]. The sense strand and antisense strand of mtDNA are named a heavy (H) strand and a light (L) strand. In human cells, mtDNA consists of 16,569 base pairs, and encodes 37 genes, including 13 polypeptides, two ribosomal RNAs, and 22 tRNAs [6,20]. One polypeptide (ND6) and eight tRNAs are located on the L strand; the other 12 polypeptides, two rRNAs, and 14 tRNAs are encoded by the H strand. mtDNA also contains a noncoding region, which is called a displacement loop (D-loop), and harbors almost all the known mtDNA replication and transcription [21]. The 13 polypeptides are the core subunit of the oxidative phosphorylation (OXPHOS) complexes I, III, IV, and V, and are essential for OXPHOS activity. Mitochondrial rRNAs and tRNAs constitute a machine for the synthesis of 13 peptides.

## 3. mtDNA Mutation and Human Diseases

mtDNA is susceptible to be attacked by oxygen free radicals, and tends to develop somatic mutations due to the lack of protection by histones [22,23]. mtDNA is located in the mitochondrial matrix, and is in close proximity to the respiratory chains [20,23], which are the main source of the reactive oxygen species (ROS). mtDNA encodes the core subunit of OXPHOS that produces the vast majority of cellular ATP. Excessive mtDNA mutations could result in the dysfunction of OXPHOS, which subsequently leads to diseases associated with mitochondrial function. In fact, many diseases have been found to be associated with mtDNA mutations, and most maternal mtDNA diseases can transmit to their offspring due to the feature of matrilineal inheritance in mtDNA [24].

Since the first human mtDNA mutation was described in 1988 [25], several mtDNA mutations and the associated mtDNA diseases have been identified. The obvious feature of mtDNA diseases is characterized by the presence of various neurological features [19]. Kearns–Sayre syndrome (KSS) and Leber’s hereditary optic neuropathy (LHON) are the early identified syndromes associated with mtDNA mutation [26,27]. KSS is associated with progressive myopathy, ophthalmoplegia, and cardiomyopathy, which is caused by single, large-scale deletions [25,26]. LHON is an optic neuropathy that is caused by mtDNA point mutations (m.3460G > A, m.11778G > A, and m.14484T > C) [27,28,29]. The point mutation of ATP6 (m.8993T > C or 8993T > G), which is the core subunit of OXPHOS protein complex V, contributes to Leigh syndrome (LS), which is also known as subacute necrotizing encephalomyelopathy [30,31]. Myoclonic epilepsy with ragged-red fibers (MERRF), which is a severe neuromuscular disorder accompanied by symptoms of myoclonic epilepsy, myopathy, dementia, or ataxia, is caused by the point mutation of tRNA [32,33].

Additional, mtDNA mutations are associated with other human diseases, including diabetes, Alzheimer’s disease (AD), Parkinson’s disease (PD), and cancer. Diabetes is one of the most common chronic disorders. mtDNA point mutations (m.3242A > G) and the 10.4-kb deletion of mtDNA are associated with diabetes and deafness, and the mutations are maternally inherited [34,35]. It is hypothesized that mtDNA mutations accumulate over time, which plays a central role in the process of aging and related neurodegeneration [19]. In fact, there is already a lot of evidence that demonstrates that mtDNA mutations are indeed associated with aging, Parkinson’s disease, and Alzheimer’s disease. Recent evidence suggests that dysregulated mitochondrial dynamics and mutations caused by mtDNA replication can lead to aging, and the increasing mtDNA mutation rates increase the aging rate and provide an aging clock [36]. A high level of deleted mtDNA has been found in the substantia nigra neurons of patients with aging and Parkinson’s disease [37]. Parkinson’s disease is a neurodegenerative disease that is characterized by the loss of dopamine neurons in the substantia nigra of the brain and the accumulation of α-synuclein [38]. Alzheimer’s disease, another neurodegenerative disease, is associated with heteroplasmic mtDNA mutations [39]. In addition, tumors and mtDNA mutations are also inextricably linked. mtDNA mutations contribute to tumorigenicity. ND3 gene mutation (m.G10398A) had been found to increase the risk of invasive breast cancer in African-American women [40]. Further data demonstrate that both germ-line and somatic mtDNA mutations contribute to prostate cancer, and about 11% of all prostate cancer patients harbored mt-CO1 (mitochondrially encoded cytochrome c oxidase I) mutations [41]. Additionally, the pathogenic mtDNA ATP6 T8993G germ-line mutation was found to generate tumors that were seven times larger than the wild type (T8993T) [41].

## 4. mtDNA Distribution

The mitochondrion is a highly dynamic double-membrane organelle that forms a well-distributed network in the majority of mammalian cell types. mtDNA is located in the mitochondrial matrix, associated with the mitochondrial inner membrane, and distributed throughout the mitochondrial network [20]. Each mitochondrion contains one or more mtDNA molecules [6]. In proliferative cells, mtDNA is replicated, separated, and distributed equally to daughter cells, which are dependent on mitochondrial dynamics. In addition, the mitochondrial membrane structure and membrane composition are also involved in mtDNA attachment and distribution [20].

### 4.1. mtDNA Distribution and Mitochondrial Dynamics

Mitochondria continuously undergo fusion and fission, which are essential for cell metabolic activities, as well as mtDNA distribution in mitochondria. Mitochondrial fusion and fission, the two opposite processes, are both mediated by large GTPases proteins, which are conserved in yeast, flies, and mammals [42]. Mitochondrial fusion is mediated by three GTPases proteins: Mitofusin 1 (Mfn1), Mitofusin 2 (Mfn2), and Optic Atrophy 1 (OPA1) [43,44]. As the feature of a double membrane, mitochondrial fusion is a two-step process requiring outer-membrane fusion followed by inner-membrane fusion [1]. Mfn1 and Mfn2 regulate the mitochondrial outer membrane fusion, and OPA1 is involved in mitochondrial inner membrane fusion [45]. A deficiency of fusion results in severe mitochondrial fragmentation and is associated with a range of human diseases [46,47]. The mutation of Mfn2 causes Charcot–Marie–Tooth disease type 2A in human, which is a common inherited peripheral neuropathy [47,48]. The dysfunction of OPA1 is associated with dominant optic atrophy (DOA), which is an optic neuropathy caused by the degeneration of retinal ganglion cells [1,49,50]. Mitochondrial fission is regulated by Drp1, a cytosolic dynamic protein, which is recruited to mitochondria from the cytosol, forms spirals around the mitochondria, and then constricts it by hydrolyzing GTP to mediate mitochondrial scission [1,51].

**Figure 1:**
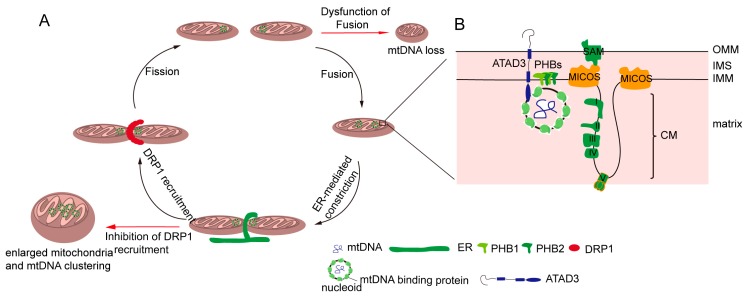
Regulation of the distribution of mitochondria DNA (mtDNA). (**A**) Mitochondrial dynamics regulate mtDNA. Mitochondrial fission and mtDNA segregation happened synchronously, and occur at the ER and mitochondrial contact site. Upon fission, the endoplasmic reticulum (ER) wraps the mitochondria, and then the cytosolic dynamic protein Drp1 is recruited to mediated mitochondria division. Blocking the fission leads to enlarged mitochondria and an mtDNA cluster. Mitochondrial fusion allows for two mitochondrial exchange substances, including mtDNA. The dysfunction of fusion leads to mtDNA deletion. (**B**) The mitochondrial inner membrane is involved in mtDNA distribution. Certain mitochondrial inner membrane proteins such as prohibitins and ATPase family AAA domain-containing protein 3 (ATAD3) are mtDNA-binding proteins. In addition, mtDNA nucleoid contacts with the mitochondrial cristae junction, and MICOS complex and Sam50, which are involved in the maintenance of the cristae structure, regulate mtDNA distribution.

Mitochondria and mtDNA are highly dynamic [52]. mtDNA are distributed throughout the mitochondrial network [53], which is important for the uniform distribution of mtDNA-encoded proteins in mitochondria. Mitochondrial dynamics greatly influence the distribution and maintenance of mtDNA [54]. A deficiency in mitochondrial fusion has a profound effect on mtDNA (Figure 1A). It has been demonstrated Mfn1 and Mfn2 conditional knock-out mice in muscle result in muscle atrophy, mitochondrial dysfunction, and severe mtDNA depletion [55]. OPA1 mediates the fusion of the mitochondrial inner membrane, and regulates cristae remodeling and cytochrome c release during apoptosis [56,57,58]. In addition, OPA1 mutations in patients lead to multiple deletions of mtDNA in their skeletal muscle [59], and one isoform of OPA1 was associated with mtDNA replication, distribution, and maintenance [60]. Mitochondrial fission also plays an essential role in mtDNA distribution. The deficiency of mitochondrial fission caused by the loss of Drp1 leads to hyperfused mitochondria and enlarged mtDNA nucleoids characterized by mtDNA accumulation [54,61,62]. Mitochondrial fusion promotes complementation between two mitochondria, including mtDNA [42,63]; mitochondrial fission separates mtDNAs into two divided mitochondria, and also contributes to a chance for a mitochondrion to re-fuse with another part of the mitochondrial network (Figure 1A). Therefore, mtDNA are distributed throughout the network by continuous fusion and fission [54].

The distribution of mtDNA is tightly interlinked with the dynamics of mitochondria, but the mechanisms of mtDNA distribution throughout the mitochondrial network are poorly understood. Recent evidence shows the close proximity between mtDNA and the sites of Drp1-dependent mitochondrial fission, which is highly conserved in yeast and mammalian cells [61,64,65]. In yeast and mammalian cells, mitochondrial division occurs at the endoplasmic reticulum (ER) and mitochondria contact sites (Figure 1A), in which the ER wraps around the mitochondria; then, Drp1 is recruited and assembled around mitochondria [66,67]. Moreover, the majority of ER-linked mitochondrial division events occur adjacent to nucleoids [20,65]. Following mtDNA replication, ER-linked mitochondrial fission occurs between the replicated mtDNAs, which locate at newly generated mitochondrial tips after scission [53,64,65]. Localizing mtDNA to the newly formed mitochondrial tips could transport mtDNA to the distal parts of cell, and further fuse with other mitochondria to drive mtDNA distribution. The mechanism can explain how mtDNA is equivalently distributed in cells and how mtDNA is distributed into mitochondria following mtDNA replication.

### 4.2. mtDNA Distribution and Inner Membrane Structure

The structure of the inner mitochondrial membrane (IMM) is divided into two morphologically and presumably functionally distinct subdomains: the inner boundary membrane (IBM), which is closely opposed to the outer mitochondrial membrane (OMM), and the cristae membrane (CM), which protrudes into the matrix [20,68]. The IBM comes into close contact with the OM by the protein transport complexes [68,69,70]. The CM is formed by the invaginations of the IBM, and is enriched in respiratory chain complexes and some small molecules and metabolites [68,71]. There is another substructure of the inner membrane—the cristae junction—that connects the IBM with the CM [72,73]. It has been reported mtDNA is associated with the IMM, and mtDNA is frequently observed intertwined into cristae [20]. Therefore, there may be several IMM factors regulating mtDNA distribution. Indeed, it has been found that the MICOS (mitochondrial contact site and cristae junction organizing system) locates at the cristae junction and is involved in regulating the inner mitochondrial membrane cristae junction [71,74,75]. In yeast, MIC60 (Fcj1) and Mic10 (Mos10), two key components of the MICOS, regulate mtDNA nucleoid size and distribution [76]. Deficiencies in the two proteins result in the formation of large mtDNA nucleoids and giant spherical mitochondria [76]. Consistently, we have found that MIC60 (IMMT) knockdown led to alterations of mitochondrial tubular morphology to giant spherical mitochondria and the disorganization and clustering of nucleoids in mammalian cells (Figure 1A) [77]. Sam50, a MICOS-interacting protein in mammalian cells, is located at the outer mitochondrial membrane [78]. The loss of Sam50 results in the disorganization of cristae and large spherical mitochondria, and also leads to enlarged mtDNA nucleoids, which protect mtDNA from clearance by mitophagy [79]. However, how the mitochondrial inner membrane regulates mtDNA organization and distribution remains unknown. It has been hypothesized that cristae junctions contribute to maintaining proper internal membrane compartmentalization, and the loss of these junctions leads to clustering and the missegregation of mtDNA nucleoids due to the loss of proper compartmental localization of the mtDNA within the mitochondrial tubules [71].

### 4.3. mtDNA Distribution and Cholesterol

Cholesterol is a composition of lipid rafts, and contributes to being a dynamic glue that keeps the raft assembly together [80,81]. Recent data demonstrate that the human mtDNA–protein complex colocalizes with the cholesterol-rich membrane [82]. Additional, cholesterol is also rich at the site of the ER-associated mitochondrial membrane (MAM), which is involved in mtDNA distribution and segregation [20,83,84]. Thus, it is possible that cholesterol is associated with the distribution of mitochondrial nucleoids. ATAD3 (ATPase family AAA domain-containing protein 3), locating at the mitochondrial inner membrane, is colocalized with mitochondrial nucleoids in mammalian cells by binding to the D-loop of mtDNA (Figure 1B) [85,86]. A deficiency of ATAD3 in cells results in the disorganization of mitochondrial nucleoids, which is also found in the mouse model and in patients with pathogenic mutations in ATAD3 [87,88]. Furthermore, ATAD3 is involved in regulating cholesterol metabolism [87,88]. Therefore, it seems that ATAD3 regulates mtDNA maintenance by regulating cholesterol metabolism.

## 5. mtDNA Release and Inflammasome

mtDNA locates in the mitochondrial matrix under normal conditions, but when apoptosis occurs, mtDNA could be released into the cytoplasm of the cell. It has been reported that mtDNA release is dependent on the NALP3 (also called NLRP3, NLR family pyrin domain containing 3) inflammasome and the production of ROS [89]. Nakahira et al. found that upon treatment with lipopolysaccharide (LPS) and ATP, wild type macrophages could produce ROS to activate the NALP3 inflammasome, which leads to the release of mtDNA (but not nuclear DNA) into the cytoplasm and causes the aggregation of mtDNA. In NALP3-deficient cells, LPS and ATP-induced mtDNA release are inhibited, although mitochondrial ROS production was not affected [89]. Thus, NALP3 is critical for mtDNA release, but how mtDNA release into the cytoplasm is still obscure. During apoptosis, the BAK-BAX play a major role in the mitochondria-mediated apoptotic pathway [90]; BAK/BAX form oligomers in the mitochondrial outer membrane and alter the permeability of the outer membrane, which result in the release of apoptotic factor cytochrome c [91,92]. The release of mtDNA is performed by a single discrete point rather than being dispersed throughout the cytoplasm and is constricted, indicating that the release of mtDNA is limited due to the presence of certain obstacles in the mitochondrial inner membrane. McArthur et al. found that when BAK/BAX is activated, cytochrome c is released outside the mitochondrial outer membrane; then, the mitochondrial network is destroyed, and the BAK/BAX oligomers are gathered and form large pores at the mitochondrial outer membrane [93]. Large pores allow the extrusion of the mitochondrial inner membrane carrying mtDNA into the cytoplasm [93,94]. In addition, Riley et al. reported that during apoptosis, BAK/BAX-mediated mitochondrial outer membrane pores gradually widen, and the mitochondrial inner membrane permeability changes, allowing mtDNA to pass through the mitochondrial inner membrane and release into the cytoplasm [95]. Importantly, mtDNA release could trigger the extracellular innate immune cGAS-TMEM173 (STING) pathway and secrete type-I interferon [96,97]. Thus, mtDNA release is highly associated with inflammation. 

## 6. mtDNA Elimination

In most types of cells, wild-type mtDNA or mutant mtDNA could be eliminated by mitophagy, which is a selective pathway to degrade damaged mitochondria. Here, we mainly discuss the elimination of paternal mtDNA. 

The most prominent feature of mtDNA is a maternal inheritance, which means that the mtDNA of offspring is inherited solely from the mitochondria of the oocyte [6,98]. Some human diseases caused by mtDNA mutations are maternally inherited. Maternal inheritance is an almost universal feature of eukaryotes, but the mechanism of paternal mtDNA clearance vary in different organisms. The “simple dilution model” has long been used to explain maternal inheritance. In this model, the copy number of paternal mtDNA is lower than that of maternal mtDNA, and mtDNA is simply diluted away by the excess of oocyte mtDNA, and consequently is hardly detectable in the offspring [98,99]. However, recent studies have found that paternal mitochondria containing mtDNA were selectively eliminated, either before or after fertilization, to prevent paternal mtDNA from transmiting to the next generation [98].

### 6.1. Endonuclease G-Mediated Degradation of Paternal mtDNA

**Figure 2:**
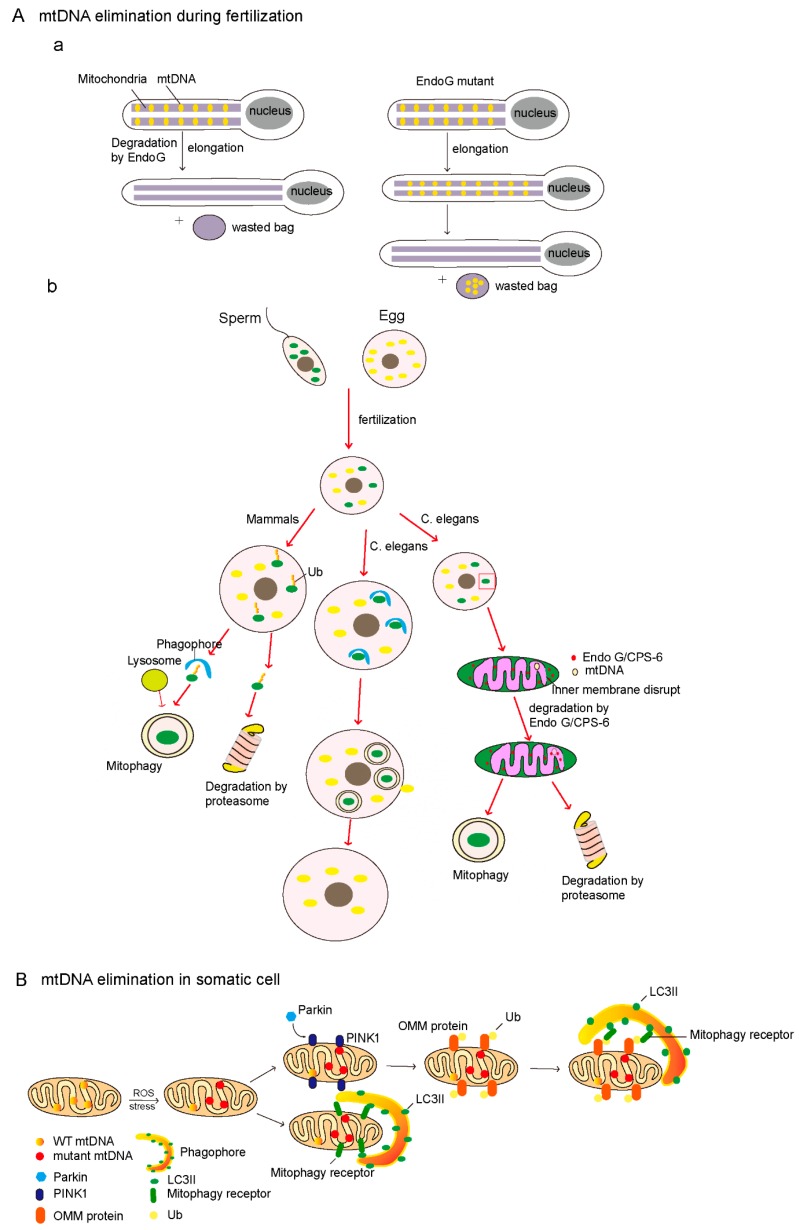
Mechanisms of mtDNA elimination. (**A**) MtDNA elimination during fertilization. (**a**) Mechanism of mtDNA elimination in *Drosophila melanogaster*. Pre-fertilization, the mtDNA of sperm in flies are deleted by endonuclease G (EndoG) during sperm elongation, as shown on the left. In the right panel, mtDNA could not clear in EndoG mutant cells during sperm elongation, but it will be deleted by another mechanism, in which mtDNA is sequestered into a waste bag and then is excluded in vitro from sperm cells. (**b**) Paternal mtDNA clearance after fertilization. In mammals (left panel), the paternal mitochondria are labeled with ubiquitination and then degraded by the lysosome or proteasome after fertilization. In *C. elegans* (middle panel), after fertilization, the paternal mitochondria are quickly wrapped by autophagic vacuoles after fertilization and degraded by the lysosome; or the mitochondrial inner membrane of paternal mitochondria loses their integrity and EndoG/CPS-6 (CED-3 protease suppressor-6) relocates from the intermembrane space to the matrix to degrade paternal mtDNA (right panel), and the paternal mitochondria are then degraded by autophagy or the proteasome machine. (**B**) The putative mechanism of degradation mtDNA in somatic cells. Mitochondrial DNA is damaged by exogenous stimulation or reactive oxygen species (ROS). Upon reaching a certain threshold, damaged mitochondria are recognized and selectively degraded by mitophagy depending on the PINK1 (PTEN induced kinase 1)–Parkin pathway or not.

DeLuca et al. showed that the mtDNA in *Drosophila* is eliminated to ensure mature spermatozoa lacking mtDNA during spermatogenesis. They found that the mitochondria of mature *Drosophila* sperm lack mtDNA, and two processes are required for clearing mtDNA during spermatogenesis [15,16]. mtDNA are gradually degraded from the sperm cells and move from the head to the tail during spermatogenesis, and mtDNA are largely cleared when the sperm are fully elongated [15]. During this process, mitochondrial endonuclease G (EndoG) is required for the degradation of paternal mtDNA [14,15,16,17]. EndoG is essential for paternal mitochondrial deletion, and EndoG mutations result in the persistence of mtDNA in elongated sperm [15,100]. Interestingly, in EndoG mutants, persisting mtDNA can be cleared by the other mechanism during the individualization stage, in which mtDNA and cellular debris are sequestered into a waste compartment that is extruded from the sperm body [15,101]. Consistently, CPS-6 (CED-3 protease suppressor-6), a mitochondrial endonuclease G in *C. elegans*, is essential for paternal mtDNA clearance [17]. The paternal mitochondria rapidly lose their inner membrane integrity in the fertilization of *C. elegans.* After fertilization, the CPS-6 relocates from the intermembrane space of the paternal mitochondria to the mitochondrial matrix to promote paternal mitochondrial mtDNA clearance (Figure 2A). CPS-6 deletion delays mitochondrial inner membrane rupture, the autophagosome enclosure of paternal mitochondria, and paternal mitochondrial elimination [17]. In addition, CPS-6 was originally recognized as an apoptotic nuclease that transferred from mitochondria to the nucleus during apoptosis, mediating chromosome breaks [102,103]. Together, endonuclease G plays a conserved role in paternal mtDNA clearance. 

### 6.2. Mitophagy-Mediated Degradation of Paternal mtDNA

Mitophagy selectively degrades damaged mitochondria, and is thought to mediate degradation of the paternal mitochondria during embryonic development [18,104,105]; certainly, mtDNA is cleared during this process. Recent findings have shown that fertilization triggers selective autophagy to prevent the transmission of paternal mitochondrial DNA to progeny, and abnormal autophagy leads to embryonic heterogeneity [18,105]. Sutovsky et al. found that autophagy and the ubiquitin–proteasome system contributed to sperm mitophagy after mammalian fertilization (Figure 2A) [18]. In rhesus monkey and pig cases, the paternal mitochondria in fertilized eggs are modified with ubiquitin, and then selectively eliminated by the proteasome or lysosome [18]. The treatment of proteasome inhibitors such as MG132 or lactacystin could block the degradation of paternal mitochondria [18,106]. On the other hand, the lysosomotropic agent ammonium chloride treatment causes the retardation of paternal mitochondrial degradation in bovine fertilized eggs [107]. However, the precise mechanism of paternal mtDNA degradation is still unclear. There are at least three putative pathways participating in clearing sperm mitochondria by autophagy and the ubiquitin–proteasome system [18]. (1) The first is P62 (sequestosome 1, SQSTM1), an ubiquitin-binding autophagy receptor, that binds to the ubiquitinated paternal mitochondria and interacts with LC3 (MAP1LC3B, microtubule associated protein 1 light chain 3 beta) or GABARAP (GABA type A receptor-associated protein) to deliver them to the lysosome for degradation. (2) The second pathway involves ubiquitinated proteins that could be extracted from the mitochondria and form aggresomes, which are the protein aggregates induced by HDAC6 (histone deacetylase 6); HDAC6 could transport aggresomes along the microtubules to the autophagosome for degradation. (3) Valosin-containing protein (VCP), a protein dislocase, could extract and deliver the ubiquitinated mitochondrial membrane proteins to the 26S proteasome for degradation [18]. In addition, prohibitin, a mitochondrial inner membrane protein, could be ubiquitinated and recognized by the ubiquitin–proteasome system of the fertilization egg [108,109].

Similarly, in *C. elegans*, sperm triggers mitophagy rapidly and subsequently paternal mitochondria degradation in the 16-cell stage (Figure 2A) [98,105]. Immediately after fertilization, autophagosomes were formed around the paternal mitochondria [105,110]. Then, paternal mitochondria engulfed by autophagosomes were delivered to lysosomes for degradation during early embryogenesis [98]. The knockdown or knock-out of *lgg-1*, an autophagy-related gene, results in the legacy of paternal mitochondria and mtDNA in late-stage embryos and even in larvae [110]. However, the ubiquitination of paternal mitochondria in *C. elegans* is not observed [105]. Therefore, the degradation of paternal mitochondria requires LC3-dependent autophagy, but is not on ubiquitinated mitochondria in *C. elegans*. Recently, the mitochondrial inner membrane protein, prohibitin-2 (PHB2), was found to be served as an mitochondrial inner mitophagy receptor to mediate mitophagy, which is essential for paternal mitochondrial elimination in *C. elegans* [111]. The loss of PHB2 results in the accumulation of sperm-derived mitochondria in the 64-cell to 100-cell stage. Thus, the interaction of PHB2 and LC3 could deliver paternal mitochondria to the lysosome for subsequent degradation in *C. elegans*. 

### 6.3. The Clearance of Mutant mtDNA

Each mitochondrion contains multiple copies of mtDNA that show a high mutation rate due to the ineffective repair mechanisms, which leads to the mutants of mtDNA. The individual cell has some mitochondria containing the mutant mtDNA and some that contain the wild-type (WT) mtDNA; this phenomenon is called heteroplasmy [112]. In heteroplasmic cells, the phenotype of a pathogenic mtDNA mutation is determined by the ratio of mutant and WT genomes [113,114]; if the ratio reaches a threshold, such as 90%, this causes the occurrence of diseases. The mutation of mtDNA is accumulated over time, which is implicated in a range of diseases including aging, Parkinson’s disease, Alzheimer’s disease, cancer, etc. Therefore, targeting mutant mtDNA to decrease the ratio of mutant mtDNA and WT has been considered as a therapeutic strategy [114]. Several engineered mitochondria-targeted site-specific nucleases have been used for the selective degradation of mutated human mtDNA [113], such as engineered zinc-finger nucleases [114,115], restriction enzymes [116,117], and transcription activator-like effector nucleases [115]. In the mouse model, mitochondria-targeted restriction endonucleases and TALENs (transcription activator-like effector nucleases) were used to prevent the transmission of mutated mtDNA to offspring [118]. These strategies are expected to be applied to prevent human diseases caused by mtDNA mutations. In addition, there must be a pathway for clearing mutant mtDNA in living organisms. In the somatic cells of *Drosophila melanogaster,* mutant mtDNA could be eliminated by mitophagy, and the stimulation of autophagy, activation of the PINK1/Parkin pathway, or decreased levels of mitofusin result in a selective decrease of mutant mtDNA (Figure 2B) [113]. However, how human cells eliminate mutant mtDNA is still obscure, which is a key scientific issue and need to be further explored.

## 7. Perspectives

mtDNA distribution is dependent on mitochondrial fission, which occurs at the endoplasmic reticulum (ER) and mitochondria contact sites, and mtDNA are separated into two daughter mitochondria during this process [64,65,66]. Thus, the disruption of mitochondrial fission may impair mtDNA distribution. Indeed, the absence of Drp1 results in the disorganization and accumulation of nucleoids [61]. In addition, we previously found that MIC60 (IMMT, inner membrane mitochondrial protein) and SAMM50 (SAMM50 sorting and assembly machinery component) play an essential role in regulating mitochondrial morphology and mtDNA distribution [77,79]. Enlarged mitochondria and accumulated mtDNA nucleoids were displayed in the absence of MIC60 or SAMM50 [77,79], because the SAMM50–MIC60 axis regulates mitochondrial membrane contact and cristae organization [74,119,120]. Moreover, mtDNA nucleoids are often in close vicinity to mitochondrial cristae. These findings suggest that the mitochondrial cristae structure may be important for the distribution of mtDNA, but how mitochondrial cristae modulate mtDNA localization remains obscure, and needs to be further explored. 

Many research studies have focused on the mechanism of the paternal mtDNA clearance in sperm or in fertilized eggs. Mitochondrial protease EndoG, the ubiquitination system, and mitophagy have been reported to play an important role in the degradation of paternal mtDNA [18,98]. However, the mechanism of mtDNA elimination in normal cells is not well understood. The PINK1/Parkin-mediated mitophagy pathway has been found to be involved in the clearance of mutant mtDNA in *Drosophila* [115]. mtDNA is encapsulated by several proteins, including Prohibitin1, Prohibitin2, TFAM, PLOG, etc., which may serve as regulators of the elimination of mutant mtDNA. Indeed, the paternal mtDNA of fertilized eggs could be eliminated by PHB2 (prohibitin-2)-mediated mitophagy [121]. In addition, we found that Sam50 depletion-induced mtDNA clustering could protect mtDNA from elimination by PINK1–Parkin-mediated mitophagy [79], suggesting that mtDNA distribution is associated with mtDNA elimination. Together, we proposed that certain mitophagy receptors may specifically recognize the mitochondria containing damaged mtDNA and mediate mtDNA elimination. Ultimately, future studies on the identification of mitophagy receptors for mtDNA elimination will be critical for advancing our understanding of mitochondrial and its related diseases.
